# Genetic and Phenotypic Diversity of *Morganella morganii* Isolated From Cheese

**DOI:** 10.3389/fmicb.2021.738492

**Published:** 2021-11-17

**Authors:** Lorenz Timo Ryser, Emmanuelle Arias-Roth, Vincent Perreten, Stefan Irmler, Rémy Bruggmann

**Affiliations:** ^1^Interfaculty Bioinformatics Unit and Swiss Institute of Bioinformatics, University of Bern, Bern, Switzerland; ^2^Agroscope, Bern, Switzerland; ^3^Institute of Veterinary Bacteriology, University of Bern, Bern, Switzerland

**Keywords:** *Morganella morganii*, biogenic amines, lysine decarboxylase, antibiotic resistances, histamine, cadaverine, putrescine, trehalose

## Abstract

The bacterium *Morganella morganii* can produce the biogenic amines (BA) cadaverine, putrescine, and histamine *in vitro* and is responsible for high histamine concentrations in fish products. These BA can have toxic effects upon ingestion and are undesired in food. The purpose of this study was to characterize the phenotype and genotype of 11 *M. morganii* isolated from cheese in regard to the BA formation. In addition, we investigated the phylogeny, trehalose fermentation ability, and antibiotic resistance of the cheese isolates. To do so, we sequenced their genomes using both long and short read technologies. Due to the presence of the trehalose operon and the ability to ferment trehalose, the cheese isolates can be assigned to the subsp. *sibonii*. Comparative genomics with public available *M. morganii* genomes shows that the genomes of the cheese isolates cluster together with other subsp. *sibonii* genomes. All genomes between subsp. *morganii* and subsp. *sibonii* are separated by an average nucleotide identity (ANI) of less than 95.0%. Therefore, the subspecies could represent two distinct species. Nine of the strains decarboxylated lysine yielding cadaverine *in vitro*. This metabolic activity is linked to a previously unknown gene cluster comprising genes encoding a lysine-tRNA ligase (*lysS*), an HTH-transcriptional regulator (*argP*), a cadaverine-lysine antiporter (*cadB*), and a lysine decarboxylase (*cadA)*. The formation of putrescine is linked to the *speF* gene encoding an ornithine decarboxylase. The gene is disrupted in five strains by an insertion sequence, and these strains only exhibit a weak putrescine production. Antimicrobial susceptibility profiling revealed that all cheese strains are resistant to tetracycline, chloramphenicol, tigecycline, colistin, and ampicillin. These phenotypes, except for colistin which is intrinsic, could be linked to antimicrobial resistance genes located on the chromosome.

## Introduction

*Morganella morganii* (formerly *Proteus morganii*) is a facultative anaerobic Gram-negative bacterium (Janda and Abbott, 2015). The bacterium is divided into two subspecies (Jensen et al., 1992). Trehalose fermenting strains are designated as *M*. *morganii* subsp. *sibonii*. The strains unable to ferment trehalose are named *M*. *morganii* subsp. *morganii*. The bacterium is present in the environment and the gastrointestinal tract of mammals and reptiles (Janda and Abbott, 2015). *M. morganii* was also found in fish products, where it produces the biogenic amine (BA) histamine (Kim et al., 2003; Kanki et al., 2007). Histamine can provoke allergic-like reactions upon consumption and is therefore undesired in food (Linares et al., 2011). In addition, various strains can decarboxylate ornithine (ODC+) and/or lysine (LDC+) to form the BA putrescine and cadaverine, respectively (Janda et al., 1996). Because cadaverine and putrescine enhance the toxicity of histamine, they are also undesired in food (Linares et al., 2011). *M*. *morganii* was also detected in cheese and the biogenic amine formation of this species could therefore have an impact on the cheese quality (Amato et al., 2012; Coton et al., 2012). The gene encoding the histidine decarboxylase of *M. morganii* was cloned and characterized by Kanki et al. (2007). Furthermore, de las Rivas et al. (2007, 2008) cloned two genes encoding ornithine decarboxylases and showed that the gene products decarboxylate ornithine. In contrast, the genetic elements responsible for cadaverine formation in *M. morganii* have not been described.

Foodborne bacteria with antibiotic resistances are undesired in cheese because they have the potential to disseminate their resistances to human pathogenic bacteria (Huddleston, 2014). *M. morganii* possesses intrinsic resistance to oxacillin, ampicillin, amoxicillin, most of the first- and second-generation cephalosporins, macrolides, lincosamides, glycopeptides, fosfomycin, fusidic acid, and colistin (Liu et al., 2016). The bacterium is currently gaining more and more attention because isolates with acquired antibiotic resistances emerge (Liu et al., 2016).

With this study, we aim to gain knowledge about *M. morganii* isolated from dairy products. Eleven strains were isolated from cheese and sequenced using both long and short read technologies. We could link phenotypic traits regarding biogenic amine formation, trehalose fermentation and antibiotic resistances to the genomic data. In addition, we examined the phylogenetic relationships of the cheese isolates with publicly available *Morganella* genomes.

## Materials and Methods

### Bacterial Strains and Cultivation

In a routine analysis, we isolated 11 *M. morganii* strains as follows: 10 grams of cheese were homogenized in 90 mL of 40°C warm peptone water (10 g L^–1^ peptone from casein, 5 g L^–1^ sodium chloride, 20 g L^–1^ trisodium citrate dihydrate, pH 7.0) for 3 min using a stomacher (Masticator, IUL instruments GmbH, Königswinter, Germany). We plated serial dilutions of the homogenate either on ECC agar (CHROMagar, Paris, France) or on agar prepared using the protocol of improved decarboxylase medium containing 10 g L^–1^ of L-lysine (Bover-Cid and Holzapfel, 1999). After an incubation at 37°C overnight, we determined the bacterial species of individual colonies on the agar plates using a MALDI Biotyper instrument (Bruker Daltonics GmbH, Bremen, Germany).

We obtained the type strain of *M. morganii* subsp. *morganii* DSM 30164 from the German Collection of Microorganisms and Cell Cultures GmbH (Braunschweig, Germany).

The *M. morganii* strains (Table 1) were stored at −80°C in Trypticase Soy Broth (TSB) (BD, Dr. Grogg Chemie AG, Stettlen-Deisswil, Switzerland) containing 30% (v/v) glycerol and were cultivated in TSB at 37°C for 20 h under aerobic conditions.

**TABLE 1:** *Morganella morganii* strains used in this study.

**Strain**	**Isolation source**	**Chromosome size (bp)**	**Plasmid size (bp)**	**Coding sequences**	**GC%**	**GenBank accession numbers**
FAM24091	Cheese surface—Raclette	4,232,212	5,222	3,655	50.3	CP066777, CP066778
FAM24206	Cheese surface—Tilsit	4,095,319	None	3,547	50.5	CP066142
FAM24670	Cheese core—Tilsit	4,280,849	5,221	3,861	50.3	CP066140, CP066141
FAM24671	Cheese core—Vacherin Mont d’Or	4,275,237	10,567	3,867	50.4	CP066138, CP066139
FAM24672	Cheese core—Vacherin Mont d’Or	4,317,505	None	3,878	50.2	CP066137
FAM24675	Cheese core—Appenzell	4,226,155	65,233; 3,186; 9,938	3,850	50.3	CP066133, CP066134, CP066135, CP066136
FAM24676	Cheese core—Vacherin Fribourgeois	4,097,492	None	3,653	50.4	CP066132
FAM24678	Cheese core—Vacherin Fribourgeois	4,186,020	72,755	3,779	50.3	CP066130, CP066131
FAM24679	Cheese core—Gruyère	4,135,673	None	3,700	50.4	CP066129
FAM24681	Cheese core—Tilsit	4,315,749	None	3,888	50.2	CP068562
FAM24685	Cheese core—Vacherin Fribourgeois	4,209,479	none	3,731	50.4	CP066127
DSM 30164	DSMZ (Stool)	3,814,728	None	3,435	51.2	CP069157

We used API 20E strips (Biomérieux, Geneva, Switzerland) to determine the capability to ferment trehalose. To assess if the *M. morganii* cheese isolates show hemolytic activity, we streaked them on Trypticase Soy agar plates containing 5% sheep blood (bioMérieux). The plates were incubated at 37°C up to 3 days and inspected for lysed red blood cells.

### Library Preparation and Whole Genome Sequencing

We sequenced 11 *M. morganii* cheese isolates (Table 1) and the *M. morganii* subsp. *morganii* type strain DSM 30164 using Oxford Nanopore Technologies (ONT, Oxford, United Kingdom) and Ion Torrent (Thermo Fisher Scientific, Baar, Switzerland). The strain DSM 30164 was included as a control.

For library preparation, we collected the bacteria of 10 mL culture in TSB by centrifugation and extracted the DNA from the pellet using the EZ1 DNA Tissue Kit on the BioRobot EZ1 (Qiagen, Basel, Switzerland) according to the manufacturer’s instructions. The DNA concentration was determined using the Qubit DNA assay kit (Thermo Fisher Scientific).

We performed the Nanopore long read sequencing as follows: 4 μg of DNA were sheared using Covaris g-TUBES. The DNA fragments were used for the library preparation using the ligation sequencing kit SQK-LSK109 (ONT) and native barcoding expansion 1–12 EXP-NBD104 (ONT). We sequenced the libraries with a Spot-ON Flow Cell (FLO-MIN110, R10) on a MinION sequencer (ONT).

For Ion Torrent sequencing we used the Ion Xpress Plus Fragment Library Kit (Thermo Fisher Scientific) to prepare bar-coded libraries out of 1 μg of DNA according to the manufacturer’s instructions. The DNA libraries were size-selected for 400-bp fragments using E-Gel SizeSelect II Agarose Gels, 2% (Thermo Fisher Scientific). The libraries were pooled and sequenced on an Ion S5 System using Ion 530 Chips (Thermo Fisher Scientific) according to the manufacturer’s protocols.

### Genome Assembly

We performed the base calling and demultiplexing of the long reads using Guppy basecaller (v3.2.4; “–config dna_r10_450bps_fast.cfg –cpu_threads_per_caller 4 –num_callers 2 –trim_strategy dna –trim_barcodes –barcode_kits EXP-NBD104’’).^1^ Afterward, we assembled the long reads using flye (v2.3.4; –“nano-raw, –genome-size 4 m”) (Kolmogorov et al., 2019) and corrected the consensus sequence of the draft genome with long reads using minimap2 (v2.17; “-ax map-ont”) (Li, 2018), racon (v1.3.1; default) (Vaser et al., 2017) and medaka (v0.10.0; ‘‘medaka_consensus’’).^2^ Base calling of short reads was performed with the Ion Torrent Suite software 5.4 (Thermo Fisher Scientific) using standard settings. We used Trimmomatic (v0.36; “CROP: 350, HEADCROP: 30, MINLEN:10”) for a quality trimming of the reads (Bolger et al., 2014). To find plasmids which were not assembled by flye, we reconstructed additional plasmids from the Ion Torrent reads with PlasmidSPAdes (v3.12.0; default) (Antipov et al., 2016). We further polished the consensus sequence of the assembly using the short reads and the tools bowtie 2 (v2.3.4.1; default) (Langmead and Salzberg, 2012) and pilon (v1.22; “–unpaired, -vcf, –changes, –tracks”) (Walker et al., 2014). We uploaded the genomes to the GenBank database where they were annotated by the NCBI prokaryotic genome annotation pipeline (Tatusova et al., 2016). GenBank accession numbers of the whole genome sequences are listed in Table 1. The average GC-content was calculated using Quast (v4.6.0; default) (Gurevich et al., 2013). For bioinformatics analyses, we retrieved 88 *M. morganii* and 4 *Morganella psychrotolerans* genome sequences from the GenBank database (November 2020, excluding assemblies with the status “anomalous” and “genome length too small,” Supplementary Table 1). Insertion sequences (IS) of interest were classified using ISfinder (Siguier et al., 2006).

### Calculation of Average Nucleotide Identity

We calculated the pairwise whole genome average nucleotide identity (ANI) of the 104 above mentioned *Morganella* spp. genomes with fastANI (v1.32; default) (Jain et al., 2018). Heat maps were generated using the python seaborn package.^3^

### Construction of Phylogenetic Tree

We constructed a phylogenetic tree based on core genes to investigate the relationship of the cheese isolates, DSM 30164 and the 88 above mentioned *M. morganii* genomes deposited in the GenBank database (Supplementary Table 1, November 2020). The four *M. psychrotolerans* genomes served as outgroup and were included in the selection of the core genes. *M. psychrotolerans* is the most closely related species of *M. morganii* (Emborg et al., 2006). The core genes were defined and aligned with Roary (v3.13.0; “-e –mafft”) (Page et al., 2015). To avoid differences in gene prediction caused by different annotation tools, we re-annotated all 104 strains (including cheese isolates) using Prokka (v1.14.6; “-mincontiglen 200, -rfam, –addgenes”) (Seemann, 2014). The phylogenetic relationship of the alignments was inferred based on maximum likelihood using RAxML including 500 bootstrap analysis (v8.2.12; “-f a -# 500 –m GTRGAMMA”) (Stamatakis, 2014) and visualized with the ETE3 Toolkit (v3.1.1) (Huerta-Cepas et al., 2016). Branch lengths were ignored and branches with a bootstrap value below 70% were collapsed.

### Determination of Cadaverine and Putrescine Formation *in vitro*

To determine the capability to produce cadaverine and putrescine, we incubated the cheese strains in IDM broth under aerobic conditions (adapted from Bover-Cid and Holzapfel, 1999; without the addition of bromocresol purple and agar) at 37°C for 20 h. The broth was supplemented with 10 g L^–1^ L-lysine and 10 g L^–1^ L-ornithine (Merck, Darmstadt, Germany). A non-inoculated medium was used as a negative control and standard solutions of cadaverine (Sigma-Aldrich, Dr. Grogg Chemie AG, Stettlen-Deisswil, Switzerland) and putrescine (Merck, Darmstadt, Germany) served as positive controls. The cell suspension was centrifuged at room temperature with 4,000 g for 5 min.

We determined the content of biogenic amines using high-pressure liquid chromatography (HPLC). Therefore, we mixed 500 μL of the culture supernatant with 100 μL of 20 mM 1,7-diaminoheptane which served as internal standard. Then, 5 mL of extraction solution (0.1 M perchloric acid in 50% acetonitrile) was added. The mixture was centrifuged (890 × g for 10 min at 10°C). A 200 μL volume of the supernatant was then treated with dansyl chloride and analyzed using HPLC as described in Ascone et al. (2017).

### Determination of Antimicrobial Susceptibility

We determined the antimicrobial susceptibility by measuring the minimal inhibitory concentration (MIC) in cation-adjusted Müller-Hinton broth using Sensititre^®^ susceptibility MIC plate EUVSEC (Thermo Fisher Scientific). The following antibiotics were tested: sulfamethoxazole (SMX), trimethoprim (TMP), ciprofloxacin (CFX), tetracycline (TET), meropenem (MERO), azithromycin (ATM), nalidixic acid (NLX), cefotaxime (CTX), chloramphenicol (CHL), tigecycline (TGC), ceftazidime (CEF), colistin (COL), ampicillin (AMP), and gentamicin (GEN). We evaluated the sensitivity according to EUCAST (AMP, CEF, CTX, MERO, CFX, GEN, TGC, CHL, COL, TMP) (The European Committee on Antimicrobial Susceptibility Testing. Breakpoint tables for interpretation of MICs and zone diameters. Version 11.0, 2021)^4^ and Clinical and Laboratory Standards Institute (NLX, TET, SMX) (CLSI, 2020) breakpoint tables for *Enterobacterales*. We searched antibiotic resistance genes in the cheese isolate genomes with Abricate (v1.0.1; “–minid 80, --mincov 80’’)^5^ using the CARD database (v3.0.8) (Jia et al., 2017).

## Results

### Isolation, Identification, and Genome Sequencing

During routine analyses of various cheeses for the presence of *E. coli* and other coliforms using chromogenic media, individual colonies were identified as *M. morganii* using the MALDI Biotyper (data not shown).

We sequenced the genomes of 11 cheese isolates and the type strain *M. morganii* subsp. *morganii* DSM 30164 using both short read and long read sequencing technologies. This mix of sequencing technologies allowed us to assemble complete chromosomes and plasmids. The chromosome size of the cheese isolates is between 4.0 and 4.3 Mb, whereas the genome size of the DSM 30164 is 3.8 Mb (Table 1). The GC-content ranges from 50.16 to 50.45%, whereas the genome of DSM 30164 has a GC-content of 51.16% (Table 1).

### Trehalose Fermentation

**FIGURE 1:**
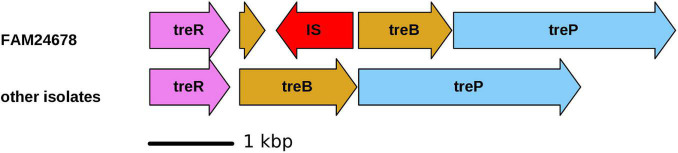
Schematic illustration of the trehalose operon of FAM24678 (not fermenting trehalose) and the other 10 cheese isolates (fermenting trehalose). *TreR*, HTH-type transcriptional regulator; *treB*, PTS system trehalose-specific EIIBC component; *treP*, trehalose 6-phosphate phosphorylase; IS, insertion sequence.

The capability to ferment trehalose is used as the criterion to classify *M. morganii* into subsp. *sibonii* (trehalose fermenter) and subsp. *morganii* (no trehalose fermenter) (Jensen et al., 1992). This phenotype is also linked to the presence of the trehalose operon (*treR*, *treB*, and *treP)* (Palmieri et al., 2020). This operon is present in the genome of all cheese isolates. All cheese isolates except FAM24678 fermented trehalose in the API assay. The *treB* gene of FAM24678 is disrupted by an IS5 family element, which likely explains the trehalose-negative phenotype of this strain (Figure 1). We identified the trehalose operon also in all other seven genomes of cluster III and in the genomes of the strains GCSL-TSO-24, H1r (both cluster II), and MMSCG (cluster IV) (see section “phylogenetic relationship”). The locus is absent in all genomes from cluster I and in the genomes of the *M. psychrotolerans* strains.

### Phylogenetic Relationship

**FIGURE 2:**
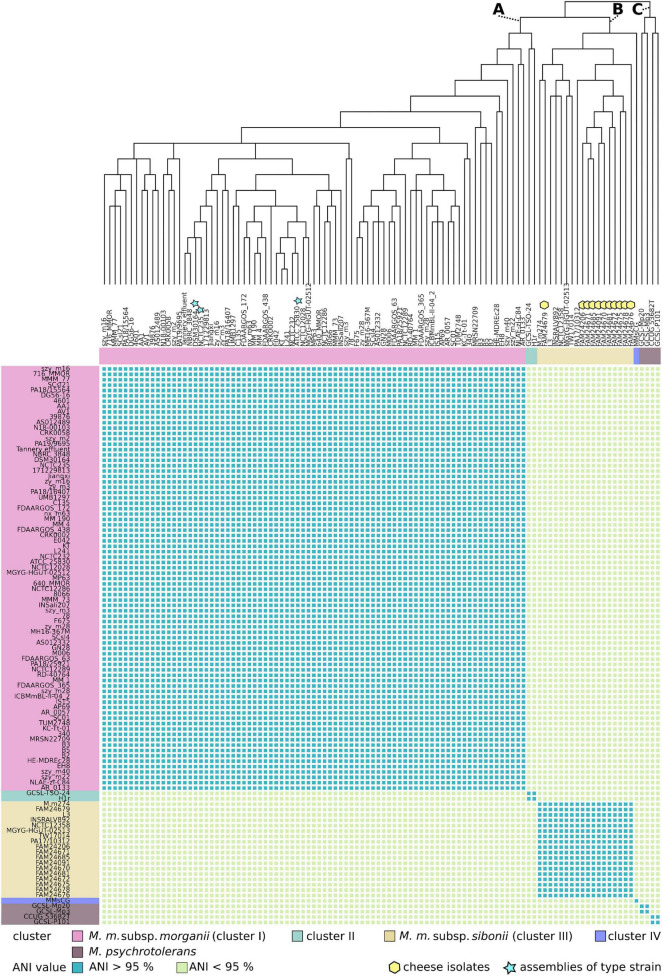
The upper part of the figure displays a phylogenetic core gene tree of 100 *M. morganii (M. m.)* and four *M. psychrotolerans* genomes. The tree was inferred based on maximum likelihood and branches with bootstrap values below 70% were collapsed. A, B, and C mark the three main branches of the tree. The heat map in the lower part of the figure shows the pairwise whole genome average nucleotide identity (ANI) values. An ANI value cut-off of 95% separates the *M. morganii* genomes into the clusters I, II, III, and IV.

The ability of the cheese isolates to ferment trehalose and the presence of the trehalose operon suggest that they belong to the subsp. *sibonii.* To gain a detailed insight into the phylogenetic relationship of the cheese isolates, DSM 30164 and 92 *Morganella* genomes from GenBank, we generated a phylogenetic tree based on 559 core genes using maximum likelihood (Figure 2).

The tree branches out into three main groups (A, B, and C in Figure 2). All strains of *M. psychrotolerans* cluster together on branch C, whereas the strains of *M. morganii* are located either on branch A or B. The cheese isolates group together and are located on branch B. The DSM 30164 strain is present in branch A and shows proximity to NCTC 235, which represents the type strain of *M. morganii* deposited in the National Collection of Type Cultures (Salisbury, United Kingdom). A boundary of 95% ANI is proposed to distinguish between bacterial species (Richter and Rosselló-Móra, 2009; Chun et al., 2018; Jain et al., 2018). When we applied an ANI cut-off value of 95%, the *M. morganii* genomes revealed four different clusters (I, II, III, and IV in Figure 2). The largest cluster I belongs to branch A and contains 79 genomes including the three assemblies of the *M. morganii* subsp. *morganii* type strain. The genomes of the strains GCSL-TSO-24 and H1r which also belong to branch A form together a small cluster (cluster II). The second largest cluster (cluster III) comprises 18 genomes including all 11 cheese isolates and belongs to branch B. This branch also includes the genome of strain MMSCG. However, the ANI value of this genome was lower than 95% when compared to the ones of branch B. In addition to the separation based on the ANI values, the mean GC-content of cluster I (51.0%) and cluster III (50.4%) genomes is statistically significant different (*P* < 0.05, unpaired two-samples *t*-test) (Supplementary Figure 1).

### Biogenic Amine Formation

Ingested histamine can provoke allergic-like reactions in humans. The toxicity of histamine is enhanced by the presence of cadaverine and putrescine (Linares et al., 2011). Therefore, bacteria producing cadaverine, putrescine, or histamine are undesirable in cheese. We determined the capability of the *M. morganii* cheese isolates to produce biogenic amines.

**FIGURE 3:**
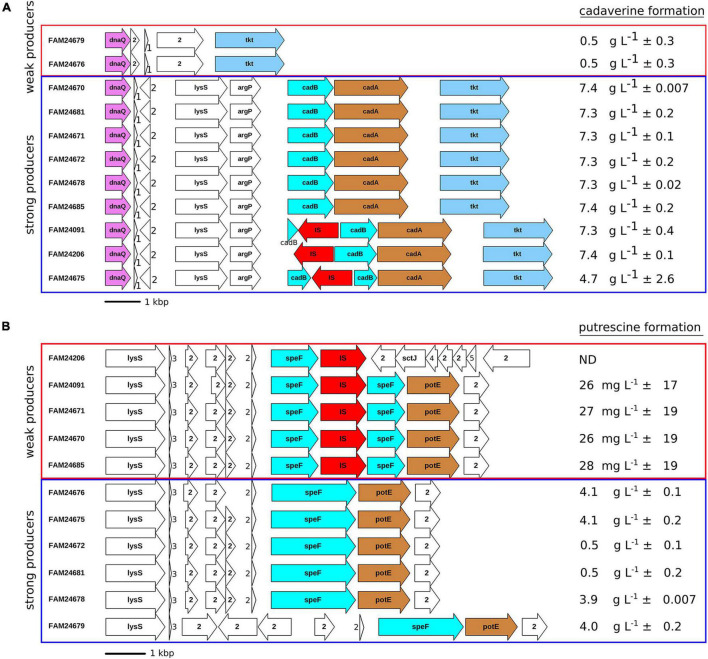
Genetic organization of loci containing the genes involved in **(A)** cadaverine formation and **(B)** putrescine formation. Arrows indicate open reading frames. *DnaQ*, DNA polymerase III subunit epsilon; *tkt*, transketolase; *lysS*, lysine-tRNA ligase; *argP*, HTH-transcriptional regulator; *cadB*, cadaverine-lysine antiporter; *cadA*, lysine decarboxylase; IS, insertion sequence; *speF*, ornithine decarboxylase; *potE*, putrescine-ornithine antiporter; *sctJ*, type III secretion inner membrane ring lipoprotein, 1: tRNA^Asp^, 2: hypothetical protein, 3: tRNA^Gly^, 4(*sctI*): type III secretion system inner rod subunit, 5 (*sctF*): type III secretion system needle filament subunit. The mean ± standard deviation of biogenic amine formation of two replicates determined by HPLC is shown (mg of biogenic amines per L broth). Rectangles mark strong (blue) and weak (red) biogenic amine producers. Some orthologs are colored in order to increase readability. ND: below limit of quantification.

All cheese isolates produced histamine (data not shown), whereas the formation of cadaverine and putrescine was strain dependent. Nine cheese isolates (FAM24670, FAM24681, FAM24671, FAM24672, FAM24678, FAM24685, FAM24091, FAM24206, and FAM24675) produced cadaverine in the range of 4.6–7.4 g L^–1^ (strong producers) and two isolates (FAM24679 and FAM24676) produced a maximum of 0.5 g L^–1^ cadaverine (weak producers) when incubated in broth containing L-lysine (Figure 3A), respectively. The strong cadaverine producer possess a gene that is annotated as lysine decarboxylase *ldcC*. The translated amino acid sequence of this gene is identical among the strong producers. Because the percent identity of the protein sequence is higher to the *Escherichia coli* inducible lysine decarboxylase CadA (68.5%, Swiss-Prot accession number P0A9H3) than to the *E*. *coli* LdcC (61.8%, UniProtKB accession number P52095), we refer to the lysine decarboxylase gene of *M. morganii* as *cadA* in this report.

The genomic region surrounding *cadA* revealed the three genes *lysS*, *argP*, and *cadB* (Figure 3A). The result of BLASTp searches of the protein sequences against the UniProtKB database propose that the genes encode for a lysine-tRNA ligase (LysS), an HTH-transcriptional regulator (ArgP), and a cadaverine-lysine antiporter (CadB) (data not shown). The *cadB* gene of FAM24091, FAM24206, and FAM24675 is disrupted by an IS3 element. Interestingly, the disruption of this gene did not affect the capability to produce cadaverine as these three strains still exhibited strong production of cadaverine.

When L-ornithine was present in the medium, six of the cheese isolates (FAM24676, FAM24675, FAM24672, FAM24681, FAM24678, and FAM24679) produced putrescine in the range of 0.5–4.2 g L^–1^ (strong producers) (Figure 3B) and five cheese isolates (FAM24206, FAM24091, FAM24671, FAM24670, and FAM24685) produced less than 30 mg L^–1^ putrescine (weak producers). de las Rivas et al. (2007, 2008) showed that both genes *speF* and *speC* encode an ornithine decarboxylase in *M. morganii* and are involved in putrescine formation. All study strains possess both *speF* and *speC*. While the nucleotide sequence of *speC* is identical in all strains (data not shown), an IS3 family insertion sequence disrupts the *speF* gene in all five weak putrescine producers (Figure 3B). A putrescine-ornithine antiporter (*potE*) is located downstream of *speF* (de las Rivas et al., 2007). The antiporter is present in all but one cheese isolate. This indicates that the *potE* is not affected by the IS in *speF*. Only one weak putrescine producer (FAM24206) misses the antiporter.

### Antibiotic Susceptibility Testing and Resistance Genes

Foodborne bacteria exhibiting antibiotic resistances may act as an antibiotic resistance gene reservoir and can pass their resistances to other bacteria in the human intestinal tract (Huddleston, 2014). We performed antibiotic sensitivity tests with all *M. morganii* cheese isolates and could show that all strains exhibited resistance against tetracycline, chloramphenicol, tigecycline, colistin, and ampicillin (Table 2). Additionally, the strains FAM24679, FAM24675, and FAM24678 were resistant against trimethoprim.

**TABLE 2:** Minimal inhibitory concentrations (MIC) determined for 14 antibiotics^a^.

**Strain**	**SMX** **(512)^b^**	**TMP** **(4)**	**CFX** **(0.5)**	**TET** **(16)**	**MERO** **(8)**	**ATM** **(NA)**	**NLX** **(32)**	**CTX** **(2)**	**CHL** **(8)**	**TGC** **(0.5)**	**CEF** **(4)**	**COL** **(2)**	**AMP** **(8)**	**GEN** **(2)**
FAM24091	256	4	0.03	**64**	0.12	32	≤4	≤0.25	**64**	**4**	≤0.5	**>16**	**>64**	1
FAM24206	256	2	0.03	**64**	0.12	32	≤4	≤0.25	**64**	**4**	≤0.5	**>16**	**>64**	2
FAM24670	256	4	0.03	**32**	0.12	32	≤4	≤0.25	**64**	**4**	≤0.5	**>16**	**>64**	1
FAM24671	128	2	0.03	**32**	0.12	32	≤4	≤0.25	**32**	**2**	≤0.5	**>16**	**>64**	1
FAM24672	64	4	≤ 0.015	**64**	0.12	32	≤4	≤0.25	**32**	**4**	≤0.5	**>16**	**32**	≤0.5
FAM24675	256	**8**	0.03	**64**	0.12	64	≤4	≤0.25	**64**	**2**	≤0.5	**>16**	**>64**	1
FAM24676	256	4	0.03	**64**	0.12	64	≤4	≤0.25	**64**	**4**	≤0.5	**>16**	**>64**	1
FAM24678	128	**8**	0.03	**32**	0.12	64	8	≤ 0.25	**64**	**2**	≤0.5	** > 16**	**>64**	≤0.5
FAM24679	512	**8**	≤0.015	**64**	0.12	32	≤4	≤0.25	**32**	**4**	≤0.5	**>16**	**>64**	1
FAM24681	64	2	≤0.015	**64**	0.12	32	≤4	≤0.25	**32**	**2**	≤0.5	**>16**	**64**	≤0.5
FAM24685	256	2	≤0.015	**32**	0.12	32	≤4	≤0.25	**32**	**4**	≤0.5	**>16**	**>64**	1

*^a^SMX, sulfamethoxazole; TMP, trimethoprim; CFX, ciprofloxacin; TET, tetracycline; MERO, meropenem; ATM, azithromycin; NLX, nalidixic acid; CTX: cefotaxime; CHL, chloramphenicol; TGC, tigecycline; CEF, ceftazidime; COL, colistin; AMP, ampicillin and GEN, gentamicin.*

*^b^ Values in brackets indicate the breakpoints defined by EUCAST and CLSI. The breakpoints are expressed in μg mL^–1^. MICs higher than the breakpoints are shown in bold. NA: breakpoint not available.*

Screening of the genomes for known antibiotic resistance genes revealed that that all *M. morganii* cheese isolates possess an *ampC* gene (DHA family class C beta-lactamase), a *tet(D)* gene (tetracycline efflux transporter), a *catA* gene (chloramphenicol O-acetyltransferase), and an *acrA* gene (multidrug efflux pump subunit) (Table 3). Ruzin et al. (2005) showed that *acrA* is associated with tigecycline resistance in *M. morganii*. Interestingly, all antibiotic resistance genes are located on the chromosome and not on plasmids. *M. morganii* has an intrinsic resistance to colistin (Liu et al., 2016).

**TABLE 3:** Locus tags of the antibiotic resistance genes in *M. morganii* genomes.

**Strain**	***ampC***	***tet(D)***	***catA***	***acrA***
FAM24091	CXB74_016365	CXB74_003560	CXB74_014025	CXB74_015975
FAM24206	JC862_15795	JC862_03460	JC862_13500	JC862_15405
FAM24670	JC861_16610	JC861_03565	JC861_13950	JC861_16220
FAM24671	JC830_16625	JC830_03565	JC830_13935	JC830_16235
FAM24672	JC827_16995	JC827_03465	JC827_14395	JC827_16605
FAM24675	JC826_16240	JC826_03535	JC826_13795	JC826_15850
FAM24676	JC825_15720	JC825_03430	JC825_13320	JC825_15290
FAM24678	JC792_15990	JC792_03520	JC792_13790	JC792_15600
FAM24679	JC793_15825	JC793_03445	JC793_13730	JC793_15430
FAM24681	JC794_16980	JC794_03470	JC794_14400	JC794_16590
FAM24685	JA116_16255	JA116_03555	JA116_13885	JA116_15865

## Discussion

### Taxonomic Assignment

Since the initial description of *Morganella morganii* (*Proteus morganii)* the species has undergone several taxonomic reclassifications (O’Hara et al., 2000). In the current classification, *M*. *morganii* forms together with *Morganella psychrotolerans* the genus *Morganella* in the family *Morganellaceae* (Emborg et al., 2006). Based on trehalose metabolism, *M. morganii* is divided into two subspecies (Jensen et al., 1992). Trehalose fermenting *M*. *morganii* are designated as *M*. *morganii* subsp. *sibonii*, whereas *M. morganii* unable to ferment trehalose belong to *M*. *morganii* subsp. *morganii*. Ten out of the 11 *M. morganii* cheese isolates of this study fermented trehalose. The genome data showed that all cheese isolates possess the trehalose operon. The absence of trehalose fermentation in one strain is likely caused by the disruption of the *treB* gene of the trehalose operon by an IS element. Considering phenotypic and genotypic characteristics, all cheese isolates belong to *M. morganii* subsp. *sibonii*. Therefore, we recommend to perform both genetic and physiological characterization to determine the subspecies of new isolates to avoid wrong classifications.

The core genome-based clustering of the *Morganella* spp. genomes retrieved from GenBank and the cheese isolates confirm that *M. morganii* and *M. psychrotolerans* can be distinguished using genomic analysis. Palmieri et al. (2020) showed that the trehalose operon negative (subsp. *morganii*) and the trehalose operon positive (subsp. *sibonii*) strains group separately. Only the trehalose operon positive strain H1R clustered together with the subsp. *morganii* strains. Remarkably, in the current study, all strains between subsp. *morganii* (cluster I) and subsp. *sibonii* (cluster III) are separated by an ANI of less than 95.0%. This ANI cutoff score of less than 95.0% is typically used to separate prokaryotic organisms into distinct species (Richter and Rosselló-Móra, 2009; Chun et al., 2018; Jain et al., 2018). Consequently, this suggests that the bacteria of cluster III constitute a distinct bacterial species. The strains H1R, GCSL-TSO-24 (cluster II) and MMSCG (cluster IV) are separated by ANI of less than 95% from both cluster I and III, respectively. This could indicate that even more putative *Morganella* species exist. However, more strains belonging to cluster II and IV would be required to confirm this hypothesis. The existence of more *Morganella* species would also explain why the trehalose operon positive H1R strain does not cluster together with the other subsp. *sibonii* strains (Palmieri et al., 2020). DNA-DNA hybridization experiments of Emborg et al. (2006) also revealed that two trehalose positive strains analyzed in their study did not group together with the strains from subsp. *sibonii*. The presence of multiple clusters of trehalose operon positive strains implies that the phenotype with respect to trehalose fermentation alone is not sufficient to distinguish the clusters.

The capability of *M. morganii* to metabolize trehalose may have an impact on its growth in cheese. González-Hernández et al. (2005) demonstrated that *Debaryomyces hansenii*, which can be part of the cheese microbiome, can produce trehalose in response to salt stress. Consequently, trehalose-degrading *M*. *morganii* may benefit from this additional energy source in cheese. This could explain why all 11 cheese isolates belong to the subsp. *sibonii*.

### Formation of Biogenic Amines

Both subspecies are further divided into biogroups based on their ability to decarboxylate ornithine (ODC+) and lysine (LDC+) to the biogenic amines putrescine and cadaverine, respectively (Jensen et al., 1992). Both cadaverine and putrescine are undesirable in cheese because they may provoke toxic reactions when ingested together with histamine (Linares et al., 2011). Several studies showed that *M. morganii* can produce toxic histamine concentrations in fish products (Kim et al., 2003; Kanki et al., 2007). In our study, all *M. morganii* cheese isolates produced histamine *in vitro* (data not shown) and exhibited strain-dependent capability to produce cadaverine and putrescine *in vitro.* The ability to form biogenic amines makes this bacterium undesirable in cheese. *In situ* experiments with *M. morganii* will clarify if the cheese isolates also produce biogenic amines in cheese.

With regard to cheese, in which the carbohydrate sources become exhausted during ripening, *M. morganii* could generate energy by amino acid decarboxylation. During the decarboxylation reaction, protons are consumed (Barbieri et al., 2019) which could lead to a proton gradient across the cell membrane. In fact, this was shown by Molenaar et al. (1993) for *Lactobacillus (para)buchneri* with the decarboxylation of histidine. The resulting proton motive force could be used for ATP synthesis. Additionally, the formation of amines increases the pH in cheese, which may be a protective measure against acidic stress in the cheese environment.

### Locus Involved in Cadaverine Formation

To the best of our knowledge, the gene encoding a lysine decarboxylase has not yet been described in *M*. *morganii*. In this study, we found that the locus containing the four genes *lysS* (lysine-tRNA ligase), *argP* (trancriptional regulator), *cad*B (cadaverine-lysine antiporter), and *cadA* (lysine decarboxylase) is responsible for strong lysine decarboxylation.

*Escherichia coli* also possesses a *cad* locus that is responsible for cadaverine formation. However, the genetic organization of the *E. coli cad* locus is different from the *cadA* locus of *M. morganii* (Meng and Bennett, 1992). Upstream of the *E. coli cadB* is an additional gene *cadC* that encodes for a DNA-binding transcriptional activator and the lysine-tRNA ligase (*lysU*) is located downstream of *cadA*.

As in *E. coli*, the genes responsible for lysine decarboxylation are located on the chromosome of the *M. morganii* cheese strains. This apparently contradicts observations made by Cornelis et al. (1981) who concluded that the lysine-decarboxylating phenotype (called lysine-positive character) is plasmid-encoded. The researchers observed that the lysine-positive character could be transferred to lysine-negative strains. It is noteworthy that the GC-content of the *cadB* and *cadA* genes of the cheese isolates is 44.4 and 38.7%, respectively. This is lower than the mean GC-content of the chromosome (50.3%). This indicates that both genes may have been acquired by horizontal gene transfer and could indeed be transferred between strains.

Remarkably, the disruption of the cadaverine-lysine antiporter *cadB* in FAM24091, FAM24206, and FAM24675 did not affect the capability to produce cadaverine. We did not find any other gene that shows similarities to cadB or cadA (lysine decarboxylase). It suggests that the lysine/cadaverine antiporter function may be taken over by other transporters in *M*. *morganii*.

### *SpeF* Is Mainly Responsible for Putrescine Formation

*M*. *morganii* possess the two genes *speF* and *speC* that encode for ornithine decarboxylases (de las Rivas et al., 2007, 2008). Both genes are present in all cheese isolates of our study. However, five cheese strains showed weak putrescine production. In these strains, an IS element disrupts the *speF* gene, whereas the *speC* gene is intact. This indicates that *speF* is the gene mainly responsible for putrescine formation observed *in vitro*. The production of cheese inoculated with strains having both the intact and the disrupted *speF* will help to understand the putrescine formation *in situ*.

### Antibiotic Resistances of the Cheese Isolates

Bacteria showing antibiotic resistances are undesirable in food because they can disseminate their resistances to human pathogenic bacteria (Huddleston, 2014). Therefore, we analyzed antibiotic resistances of the *M. morganii* cheese isolates. All strains were resistant against ampicillin, tetracycline, colistin, and tigecycline. *M. morganii* possesses an *ampC* β-lactamase which makes the species naturally resistant to ampicillin (Liu et al., 2016). We detected the gene in all cheese isolates. We could link the tetracycline resistance to the tetracycline efflux transporter *tet(D)* gene (Henriques et al., 2008). The resistance to tetracycline is widespread in *M. morganii* subsp. *sibonii.* However, it can also occur in *M. morganii* subsp. *morganii* strains (Stock and Wiedermann, 1998). *M. morganii* has an intrinsic colistin resistance, which is conferred by the structure, composition and modifications of lipid A that prevents the binding of the antibiotic to the lipopolysaccharides of the outer membrane (Olaitan et al., 2014). *M. morganii*, as do all our cheese isolates, possess an AcrAB efflux pump that decreases the susceptibility to tigecycline (Ruzin et al., 2005). *M. morganii* is normally sensitive to trimethoprim (Liu et al., 2016). However, three cheese isolates were resistant against this antibiotic. We could not identify the resistance mechanism because the three strains do not possess known resistance genes described in the literature (Tsakris et al., 2007; Schultz et al., 2017) or CARD database. Furthermore, the amino acid sequence of the dihydrofolate reductase (DHFR), the target enzyme of trimethoprim, is identical to the DHFR sequence of sensitive cheese isolates (Watson et al., 2007).

The characteristic of resistance to chloramphenicol is not uniformly described in the literature. On the one hand, there are reports that *M. morganii* is sensitive to chloramphenicol (Janda and Abbott, 2015) and on the other hand, that *cat* genes conferring resistance to chloramphenicol are located on chromosomes (Chen et al., 2012; Liu et al., 2016). In our case, all cheese isolates show chloramphenicol resistance and possess a *cat* gene located on the chromosome. These findings indicate that chloramphenicol resistance is presumably more widespread than previously thought.

## Conclusion

In the present study we isolated *M. morganii* strains from different cheese samples. Our genomic and phenotypic analyses show that the isolates belong to subsp. *sibonii*. The differences in ANI values between subsp. *morganii* and subsp. *sibonii* suggest that they may represents distinct bacterial species. Various strains can metabolize trehalose, histidine, lysine, and ornithine, which serve as energy sources in cheese environments. We linked the metabolic activities to genetic loci, and, to the best of our knowledge, describe the locus involved in cadaverine formation for the first time. Antibiotic resistance profiles did not exhibit unusual resistances. Taken together, in this study we describe *M. morganii* cheese isolates both genetically and phenotypically, providing a useful resource for the field of comparative genomics. *In situ* experiments will clarify the impact of *M. morganii* on cheese quality.

## Data Availability Statement

The datasets presented in this study can be found in online repositories. The names of the repository/repositories and accession number(s) can be found below: https://www.ncbi.nlm.nih.gov/genbank/, CP066777, CP066778, CP066142, CP066140, CP066141, CP066138, CP066139, CP066137, CP066133, CP066134, CP066135, CP066136, CP066132, CP066130, CP066131, CP066129, CP068562, CP066127, and CP069157.

## Author Contributions

LR, SI, and VP conducted the experiments. LR and RB performed the bioinformatic analysis. LR, SI, and RB wrote the manuscript. EA-R critically revised and discussed the manuscript. All authors read and approved the final manuscript, conceived and designed the study, and analyzed the data.

## Conflict of Interest

The authors declare that the research was conducted in the absence of any commercial or financial relationships that could be construed as a potential conflict of interest.

## Publisher’s Note

All claims expressed in this article are solely those of the authors and do not necessarily represent those of their affiliated organizations, or those of the publisher, the editors and the reviewers. Any product that may be evaluated in this article, or claim that may be made by its manufacturer, is not guaranteed or endorsed by the publisher.

## Footnotes

1
https://community.nanoporetech.com/

2
https://github.com/nanoporetech/medaka

3
https://github.com/mwaskom/seaborn

4
http://www.eucast.org

5
https://github.com/tseemann/abricate
