# Supplementary material for: Genetic and Phenotypic Diversity of Morganella morganii Isolated From Cheese
Source: Front Microbiol. 2021 Nov 17;12:738492. doi: 10.3389/fmicb.2021.738492 (PMC8638253; doi:10.3389/fmicb.2021.738492)
Supplement: Supplementary file 1 [file Table_1.docx]

**Supplementary Table 1.** GenBank assemblies of *Morganella* *morganii* and *Morganella* *psychrotolerans* used in this study (November 2020).

| **Species** | **Strain** | **GenBank accesion** | **GC-content (%)** | **Genome size (Mb)** | **Assembly level** |
| --- | --- | --- | --- | --- | --- |
| *M. morganii* | NLAE-zl-C84 | GCA_900142745.1 | 50.90 | 3.92 | Scaffold |
|  | ATCC 25830 | GCA_006094455.1 | 51.00 | 3.89 | Complete |
|  | AR_0133 | GCA_003071325.1 | 50.84 | 4.30 | Complete |
|  | AR_0057 | GCA_002968775.1 | 51.00 | 4.14 | Complete |
|  | FDAARGOS_365 | GCA_002386305.1 | 51.00 | 4.08 | Complete |
|  | MP63 | GCA_010748915.1 | 51.06 | 4.01 | Complete |
|  | FDAARGOS_63 | GCA_000783955.2 | 51.10 | 4.00 | Complete |
|  | 171229813 | GCA_011465095.1 | 51.10 | 3.98 | Complete |
|  | N18-00103 | GCA_010365245.1 | 51.10 | 3.94 | Complete |
|  | FDAARGOS_172 | GCA_001558895.2 | 51.00 | 3.91 | Complete |
|  | NCTC12028 | GCA_900478755.1 | 51.00 | 3.91 | Complete |
|  | MGYG-HGUT-02512 | GCA_902387845.1 | 51.00 | 3.91 | Complete |
|  | DG56-16 | GCA_003573445.1 | 50.90 | 3.90 | Complete |
|  | L241 | GCA_003955965.1 | 51.05 | 3.90 | Complete |
|  | KC-Tt-01 | GCA_002891475.1 | 51.20 | 3.82 | Complete |
|  | KT | GCA_000286435.2 | 51.10 | 3.80 | Complete |
|  | Jiangxi | GCA_013378135.1 | 51.20 | 3.79 | Complete |
|  | GN28 | GCA_002946575.1 | 50.90 | 4.09 | Chromosome |
|  | NBRC 3848 | GCA_001598895.1 | 51.20 | 3.78 | Contig |
|  | FDAARGOS_438 | GCA_002588265.1 | 50.80 | 4.47 | Contig |
|  | M.m274 | GCA_011754435.1 | 50.30 | 4.29 | Contig |
|  | NCTC12286 | GCA_900453165.1 | 50.80 | 4.07 | Contig |
|  | NCTC232 | GCA_900453135.1 | 51.10 | 3.85 | Contig |
|  | HE-MDREc28 | GCA_003730795.1 | 51.20 | 3.85 | Contig |
|  | szy_m3 | GCA_014283925.1 | 51.30 | 3.70 | Contig |
|  | szy_m40 | GCA_014283965.1 | 51.10 | 4.01 | Contig |
|  | zy_m3 | GCA_014284025.1 | 51.10 | 3.75 | Contig |
|  | MM 190 | GCA_003287815.1 | 50.90 | 3.97 | Scaffold |
|  | MM 1 | GCA_003390295.1 | 51.00 | 3.88 | Scaffold |
|  | szy_m16 | GCA_014283915.1 | 51.10 | 3.68 | Contig |
|  | B3 | GCA_009389535.1 | 50.90 | 3.90 | Contig |
|  | EH8 | GCA_009647855.1 | 51.20 | 3.85 | Scaffold |
|  | 4601 | GCA_006517665.1 | 51.10 | 3.71 | Contig |
|  | szy_m22 | GCA_014284045.1 | 51.00 | 3.90 | Contig |
|  | MMM_77 | GCA_011030145.1 | 51.10 | 3.90 | Contig |
|  | MM 4 | GCA_003340585.1 | 51.00 | 3.82 | Scaffold |
|  | PA18/25921 | GCA_014050605.1 | 51.00 | 4.06 | Scaffold |
|  | nx_m63 | GCA_014283905.1 | 51.10 | 4.01 | Contig |
|  | CRK0002 | GCA_002184465.2 | 51.00 | 3.88 | Contig |
|  | szy_m28 | GCA_014283975.1 | 51.10 | 3.86 | Contig |
|  | E042 | GCA_002416605.1 | 51.00 | 3.98 | Contig |
|  | PA18/15564 | GCA_014050635.1 | 51.20 | 3.69 | Scaffold |
|  | PA18/16407 | GCA_014050625.1 | 51.20 | 3.71 | Scaffold |
|  | B2 | GCA_009389505.1 | 50.90 | 3.89 | Scaffold |
|  | MRSN22709 | GCA_000770295.1 | 50.70 | 4.24 | Scaffold |
|  | szy_m2 | GCA_014283995.1 | 51.10 | 3.95 | Contig |
|  | Tannery effluent | GCA_008728955.1 | 51.10 | 3.79 | Contig |
|  | 78 | GCA_003968785.1 | 51.10 | 3.85 | Contig |
|  | zy_m28 | GCA_014333515.1 | 50.90 | 4.10 | Contig |
|  | NCTC12289 | GCA_900453195.1 | 50.90 | 3.95 | Contig |
|  | 8066 | GCA_000966695.1 | 50.90 | 3.96 | Contig |
|  | SCsl21 | GCA_009822245.1 | 51.00 | 3.90 | Scaffold |
|  | 39876 | GCA_002180575.1 | 51.00 | 4.07 | Contig |
|  | M006 | GCA_002417235.1 | 51.10 | 4.14 | Contig |
|  | SCsl4 | GCA_009822545.1 | 50.70 | 4.15 | Scaffold |
|  | CRK0058 | GCA_002185325.2 | 50.90 | 3.94 | Contig |
|  | L3 | GCA_001006565.1 | 50.40 | 4.17 | Contig |
|  | IS15 | GCA_000530115.1 | 50.90 | 4.18 | Contig |
|  | SC01 | GCA_000307755.2 | 50.80 | 4.15 | Contig |
|  | INSali207 | GCA_001653675.1 | 51.10 | 3.81 | Scaffold |
|  | RD-40764 | GCA_003852695.1 | 51.10 | 4.08 | Contig |
|  | ICBMmBL-II-04(2) | GCA_002029935.1 | 50.90 | 4.12 | Contig |
|  | AP69 | GCA_003970205.1 | 50.90 | 4.14 | Contig |
|  | C135 | GCA_004349695.1 | 51.00 | 3.92 | Scaffold |
|  | UMB1297 | GCA_002847885.1 | 51.00 | 3.86 | Scaffold |
|  | MMM_73 | GCA_011030225.1 | 50.90 | 3.96 | Contig |
|  | PA17/10312 | GCA_014050645.1 | 50.30 | 4.04 | Scaffold |
|  | INSRALV892 | GCA_001263435.1 | 50.50 | 4.27 | Scaffold |
|  | H1r | GCA_000633515.1 | 50.10 | 4.51 | Contig |
|  | 340 | GCA_000747035.1 | 51.20 | 3.67 | Contig |
|  | TUM2748 | GCA_003176395.1 | 51.20 | 3.88 | Contig |
|  | AA1 | GCA_002077675.1 | 51.10 | 3.57 | Contig |
|  | AS012489 | GCA_010588235.1 | 51.40 | 4.04 | Scaffold |
|  | zy_m16 | GCA_014333575.1 | 50.90 | 3.85 | Contig |
|  | MMsCG | GCA_003996855.1 | 50.70 | 3.90 | Scaffold |
|  | 640_MMOR | GCA_001066745.1 | 51.00 | 3.97 | Scaffold |
|  | AV1 | GCA_002077705.1 | 51.20 | 3.65 | Contig |
|  | F675 | GCA_000752335.1 | 51.00 | 4.20 | Scaffold |
|  | NCTC12358 | GCA_900453145.1 | 50.40 | 4.21 | Contig |
|  | 716_MMOR | GCA_001066005.1 | 51.00 | 3.84 | Contig |
|  | B5 | GCA_009389515.1 | 50.80 | 3.92 | Contig |
|  | PA19/9695 | GCA_014050615.1 | 51.20 | 3.98 | Scaffold |
|  | MH16-367M | GCA_003114875.2 | 51.16 | 4.21 | Contig |
|  | AS012332 | GCA_010597625.1 | 51.10 | 4.20 | Scaffold |
|  | TW17014 | GCA_001274995.1 | 50.50 | 3.98 | Scaffold |
|  | MGYG-HGUT-02513 | GCA_902387785.1 | 50.50 | 3.98 | Scaffold |
|  | NCTC235 | GCA_900635025.1 | 51.20 | 3.82 | Complete |
|  | GCSL-TSO-24 | GCA_000950365.1 | 50.20 | 4.26 | Contig |
| *M. psychrotolerans* | CCUG 53682T | GCA_008692955.1 | 48.10 | 4.17 | Contig |
|  | GCSL-P101 | GCA_001676155.1 | 48.10 | 4.15 | Contig |
|  | GCSL-Mp3 | GCA_001676055.1 | 47.90 | 4.37 | Contig |
|  | GCSL-Mp20 | GCA_001676225.1 | 48.10 | 4.20 | Contig |
