# Supplementary figures and images for: Genetic and Phenotypic Diversity of Morganella morganii Isolated From Cheese
Source: Front Microbiol. 2021 Nov 17;12:738492. doi: 10.3389/fmicb.2021.738492 (PMC8638253; doi:10.3389/fmicb.2021.738492)

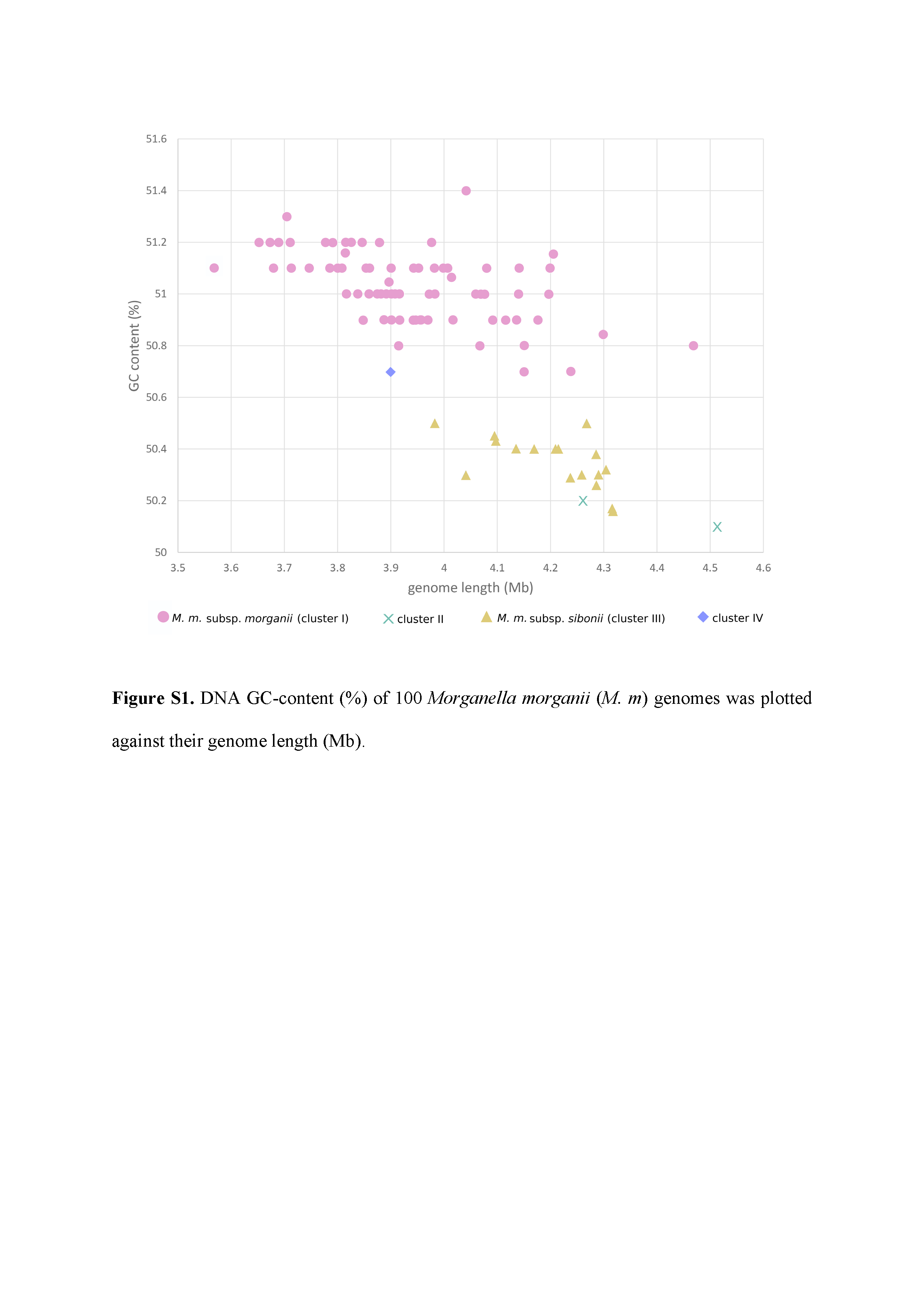

Supplement: Supplementary file 2 [file Image_1.tif]
